# Driver Mutations in Pancreatic Cancer and Opportunities for Targeted Therapy

**DOI:** 10.3390/cancers16101808

**Published:** 2024-05-09

**Authors:** Olamide T. Olaoba, Temitope I. Adelusi, Ming Yang, Tessa Maidens, Eric T. Kimchi, Kevin F. Staveley-O’Carroll, Guangfu Li

**Affiliations:** 1 Department of Surgery, University of Connecticut Health Center, Farmington, CT 06030, USA; olaoba@uchc.edu (O.T.O.); adelusi@uchc.edu (T.I.A.); minyang@uchc.edu (M.Y.); kimchi@uchc.edu (E.T.K.); 2 Department of Immunology, University of Connecticut Health Center, Farmington, CT 06030, USA; 3 Department of Surgery, University of Missouri, Columbia, MO 65212, USA; tdmnm2@umsystem.edu

**Keywords:** PDAC, targeted therapy, immunotherapy, treatment

## Abstract

**Simple Summary:**

Pancreatic cancer is highly resistant to therapies, and patients do not have a high 5-year survival rate after diagnosis. The current standard of care therapy for pancreatic cancer comprises surgery and chemotherapy. However, surgery is only beneficial in 15–20% of all cases because most patients are diagnosed at an advanced stage, and at this stage, patients’ tumors are often nonresectable. Further, the use of chemotherapy does not completely provide tumor remission. Therefore, there is a need to improve current treatment strategies or develop novel efficacious alternatives. The identification of actionable targets and the development of therapies that are specific to these targets represent a critical approach to prolonging survival in cancer patients. In this review, we discuss driver mutations in pancreatic cancer as actionable targets and provide reviewed molecules from preclinical studies and clinical trials that have been developed against these targets.

**Abstract:**

Pancreatic cancer is the sixth leading cause of cancer-related mortality globally. As the most common form of pancreatic cancer, pancreatic ductal adenocarcinoma (PDAC) represents up to 95% of all pancreatic cancer cases, accounting for more than 300,000 deaths annually. Due to the lack of early diagnoses and the high refractory response to the currently available treatments, PDAC has a very poor prognosis, with a 5-year overall survival rate of less than 10%. Targeted therapy and immunotherapy are highly effective and have been used for the treatment of many types of cancer; however, they offer limited benefits in pancreatic cancer patients due to tumor-intrinsic and extrinsic factors that culminate in drug resistance. The identification of key factors responsible for PDAC growth and resistance to different treatments is highly valuable in developing new effective therapeutic strategies. In this review, we discuss some molecules which promote PDAC initiation and progression, and their potential as targets for PDAC treatment. We also evaluate the challenges associated with patient outcomes in clinical trials and implications for future research.

## 1. Introduction

Pancreatic cancer is the sixth leading cause of cancer-related mortality. As the most common form of pancreatic cancer, pancreatic ductal adenocarcinoma (PDAC) represents up to 95% of all pancreatic cancer cases, accounting for more than 300,000 deaths annually [1]. Over the years, the estimated incidence and mortality rate for pancreatic cancer has increased. In 2023 alone, the American Cancer Society estimated that 64,050 individuals would develop pancreatic cancer, and 50,550 others would die because of pancreatic cancer. These estimated new case and mortality numbers among the American population increased by 3.7% and 2.4%, respectively, in 2024 [2,3]. Due to the lack of early diagnoses and the high refractory response to the currently available treatments, pancreatic cancer is a highly aggressive lethal malignancy, and the global 5-year overall survival rate is less than 10% [4]. In the US, the 5-year overall survival rate is 13% [2].

Genetic and epigenetic analysis of PDAC patients’ samples have revealed frequently mutated genes in PDAC. Mutations in the oncogenic KRAS gene and critical tumor suppressor genes—TP53, CDKN2A, and SMAD4—represent the canonical driver mutations that underline the initiation of the precursor lesion, and progression into PDAC. However, mutations in epigenetic regulator genes, such as ARID1A, ARID1B, SMARCA1, MLL2, MLL3, and in certain DNA repair genes (ATM, BRCA2), are less frequent in PDAC [5].

There are several factors that increase the risk of developing pancreatic cancer among people [6]. PDAC is rarely diagnosed in younger people under the age of 30, and the incidence is relatively lower in women than men globally [7,8]. In the United States, African Americans are at higher risk than Caucasians, whereas Asian Americans and Pacific Islanders have the lowest incidence [9]. The risk of developing PDAC cancer has been shown to increase exponentially as the number of first-degree relatives with a history of the disease increases [10]. These factors—age, sex, region, and family history—are the non-modifiable risk factors for PDAC. Furthermore, dysbiosis, microbial products, and the immune-suppressive activity of the microbiome contribute to the risk of developing pancreatic cancer [11,12]. Smoking, alcohol consumption, chronic pancreatitis, and obesity represent the modifiable risk factors for pancreatic cancer [13,14,15,16].

The treatment of PDAC patients is challenging. For instance, surgery represents one of the curative measures in many solid tumors. However, because patients are usually diagnosed at an advanced stage, the majority of PDAC patients present with unresectable and often metastatic disease. This therefore compromises the curative impact of surgery in PDAC patients. Nevertheless, the use of systemic chemotherapy as a surgical adjunct has improved the OS of PDAC patients over time. Recently, the increased utilization of genomic testing in advanced metastatic cancer has influenced the evolution of targeted therapy [17]. Thus, several agents that can target molecular pathways have been developed. In this review, we discuss driver mutations in oncogenes and tumor suppressors that can initiate and advance PDAC and how they can be targeted. Further, we discuss the challenges associated with the use of targeted therapy for the treatment of pancreatic cancer and future directions.

## 2. Driver Mutations, and Mechanisms of PDAC

**Figure 1:**
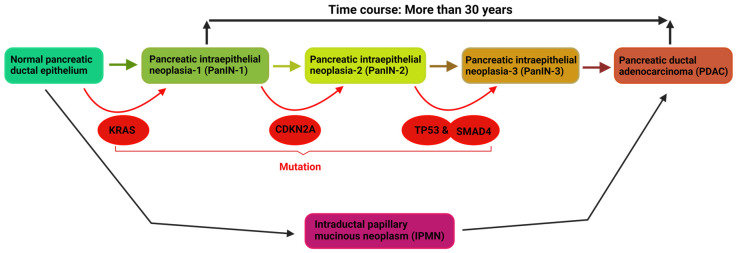
Shows the initiation and progression of pancreatic cancer. Mutation in oncogenic KRAS is sufficient to initiate the precursor lesion PanIN-1. The accumulation of mutations in the tumor suppressor genes drive PanIN-1 through PanIN-2 and PanIN-3 to PDAC. Alternatively, ductal epithelial cells may malignantly transform through IPMN to PDAC. Overall, the time course between PanIN- 1 and PDAC is more than 30 years. Peters and colleagues reported that progression from PanIN 3 to PDAC took 11.3 years and 12.3 years in men and women, respectively [22].

The term “driver mutation” in cancer was coined to explain how cancer-associated genes and DNA sequences mutate to trigger the onset, growth, and metastasis of cancer. The significance of investigating driver mutations in cancer cells is that it may help in designing drugs or therapies that can target specific mutations and therefore help nip cancers in the bud. While many genetic alterations accumulate in cancer cells throughout lifetimes, only a few of them are driver mutations. Factors that challenge the identification of driver genes include the high heterogeneity in the biochemical, histological, and mutational features of tumors [18]. One of the strategies used for identifying driver mutations in pancreatic cancer is next-generation sequencing, which has identified innumerable novel genetic aberrations. Oncogenic mutations in the KRAS gene and the loss of the tumor suppressor functions of the CDNK2A, DPC4/SMAD4, and TP53 genes are frequently observed in PDAC [19], and they are therefore referred to as the most common driver genes in PDAC [20]. Genetic analysis of clinical specimens has proven that KRAS mutations stand indefatigable as the oncogenic trigger for the onset of PDAC (Figure 1) through the precursor lesion, pancreatic intraepithelial neoplasia (PanIN1), while CDNK2, DPC4/SMAD4, and TP53 mutations are responsible for the progression, development, and invasiveness of the disease [5,20,21]. BRCA2, together with LKB1 and others, are among the infrequent mutations which are not strictly associated with the onset and progression of PDAC, but are also associated with other cancers.

KRAS (Kirsten rat sarcoma) oncogenic mutation represents the hallmark of all mutations that occur in PDAC, and it is frequently located on loop P, which is responsible for the stabilization of the active state of the nucleotide [23]. It is the most mutated gene in all of the Ras family and therefore drives oncogenesis in most cancer cells [24,25]. The most reported single-based missense point mutations are on codon 12 (G12C), codon 13 (G13), and codon 61 (Q61), representing 98% of total cases [26]. More than 90% of pancreatic cancer patients have KRAS mutations [27]. The most common point mutations reported in PDAC include G12C (1.7%), G12D (40.3%), and G12V (27.7%), with G12D (40.3%) being the most frequently mutated (Table 1) [28]. KRAS mutation triggers the onset/early event of PDAC, a condition called pancreatic intraepithelial neoplasia 1 (PanIN-1). Progression from PanIN-1 to PDAC is due to the accumulation of mutations in tumor suppressor genes, such as CDNK2A, TP53, and SMAD4 [5].

## 3. Targeting Oncogenic Signaling Pathways for PDAC Treatment: KRAS

The KRAS (a member of the Ras superfamily or Ras-like GTPase) gene belongs to the group of GTP-binding proteins that expresses a transduction GTPase activities, and which fluctuate between GDP and GTP-bound states [23]. This molecular switching protein can also be activated by guanine nucleotide exchange factors (GEFs), causing it to interact with various effectors in their GTP-bound state [35]. Importantly, it is only in the GTP-bound state that KRAS can bind and activate proteins like RAF-kinases, PI3K, and RaIGDS (Ral guanidine dissociation stimulator), which are collectively referred to as effector proteins [36]. The activation paradigm includes the oligomerization of the receptor followed by the activation of kinase activity and transphosphorylation of the catalytic domains [37]. The SH2 (sequence homology 2) domain of the receptor is an adaptor that recruits GEFs like SOS-1 (son of sevenless homolog 1) to the cell membrane [38]. GEFs then bind to Ras protein to effect the change in conformation that triggers an exchange of GDP to GTP. The downstream effect of this conformational alteration is the recruitment of various downstream signaling pathways, such as the canonical Raf/Mek/Erk, PI3K/Akt, and Ral-GEF pathways [39,40]. Restoration to the inactive state is mediated by GTPase-activating proteins (GAPs) that increase the weak intrinsic GTPase activity fivefold to inhibit KRAS activities [41,42]. On the other hand, oncogenic RAS mutants possess impaired and reduced GAP activity, which underscores permanent KRAS activation and signaling [43]. There are hotspot mutations in Ras that drastically impair GAP-mediated GTPase hydrolytic activity, leading to constitutive Ras activation, which resultantly drives cell transformation and tumor initiation in various cancer models [44]. When the germline mutations of Ras aggravate aberrant activation of their downstream MAPK (mitogen-activated protein kinase) signaling pathway, it results in RASopathies (developmental disorders) in patients. In relation to KRAS, new members of the Ras subfamily discovered through sequence homology screening possess different functions; these members include HRAS and NRAS [45]. Others include DIRAS subgroups (DIRAS-1, DIRAS-2, and DIRAS-3), which have been discovered to be tumor suppressors and are all downregulated in cancer scenarios [46,47,48].

RAF/MEK/ERK signaling is the first well-known identified Ras effector pathway involved in the mitogenic signaling of TKR, following a path of differentiation, growth, inflammation, and apoptosis [38]. Raf is a member of the serine/tyrosine kinase family, which includes Raf1, A-Raf, and B-Raf. The members of this family bind the effector region of the RAS-GTP complex to mediate the translocation of the Raf protein into the membrane [37,39]. Research has discovered that Ras activated either by point mutation (G12V) or GTP-binding could interact with Raf-1 through GMP-PNP (Guanylyl-imidodiphosphate). However, the Ile35Ala effector domain mutation of Ras does not interact with Raf-1. Through protein kinase-orchestrated phosphorylation and the activation of effector proteins, MAPK then phosphorylates its downstream effectors ERK1 and ERK2 (extracellular signal-regulated kinase 1 and 2) [40]. ERK, as a kinase, therefore, activates nuclear transcription factor and other kinases, which include EIK-1 (eukaryotic initiation factor 2 alpha kinase 1) and protein C-Ets-1 and C-ETS2 [49]. Signal responses that promote cell survival and apoptosis through various triggering factors, such as c-JNK (c-Jun N-terminal kinases), SAPK (stress-activated protein kinase), and NF-κB (nuclear factor kappa-light-chain-enhancer of activated B cells), also activate MAPK, otherwise called MEK.

The other well-studied Ras effector family is the PI3Ks (phosphoinositide 3-kinases). The binding of a ligand to the RTK marks the initiation of PI3K activation, followed by the dimerization and autophosphorylation of RTK, which ultimately leads to its interaction with the PI3K effector through the SH2 domain [50]. GRB2 (an adaptor of PI3K) binds the RTK at its phosphor-YXN motifs, followed by the binding of GRB2 to the SOS to activate it, and enables SOS-triggered RAS activation. RAS activation activates p110 of p85, while the tumor suppressor phosphatase and tensing homology (PTEN) dephosphorylates PIP3 to PIP2. Through phosphoinositide-dependent kinase (PDK1) and Akt, which belongs to the PH domains, PIP3 binds to induce intracellular signaling [51]. The association between PI3K and the RAS-signaling pathway is embedded in the promotion of cell growth and their highly correlated oncogenic signaling [49]. That is, PI3K mediates Ras-orchestrated proliferation and cell survival [52]. The activation of PI3K allows the formation of PIP3 (phosphatidyl Inositol 3, 4, 5-triphosphate) from PIP2 (phosphatidylinositol (4,5)-biphosphate), and PIP3 binds to Akt/PKB at the PH (Pleckstrin homology) domain to simulate the phosphorylation of proteins that drive cell growth, cell cycle, and survival [49].

### Targeting KRAS: Clinical and Therapeutic Investigations

KRAS is well-known as the most mutated oncogene in PDAC, and until the emergence of sotorasib and other medications (G12C inhibitors that covalently bind the G12C mutant protein switch-II pocket), it was considered to be an “untargetable” or “undruggable” oncogene for decades [53]. However, while these G12C inhibitors have demonstrated significant therapeutic efficacy in NSCLC, their efficacy is limited in PDAC due to the low frequency of G12C mutants in PDAC [53]. Following the long-term belief that KRAS pockets were undruggable due to (1) the architecture of the pockets, with their smooth and featureless surface that makes it difficult for small molecule drugs to bind for effective inhibition [54], (2) their high affinity for GTP, which makes it challenging for small molecule-inhibiting drugs to disrupt them without tampering with other important cellular components, (3) the high degree of conformational flexibility that makes it difficult for designed drugs to stabilize it, (4) the interaction of NEFs (Nucleotide Exchange Factors) with GDP that dampens the effective therapeutic efficacy of the targeting drugs due to the switching of the GDP to GTP that triggers KRAS activation [55], (5) the difficult SOS targeting due to its role in the switching of KRAS’s GDP and GTP modes and its role in other essential cellular processes, and lastly, (6) due to the toxicity/off-target effective concerns that emanate from targeting KRAS without altering its normal function in healthy cells [56,57,58,59,60], it has been well-shown that the KRAS G12C mutant is druggable [57,61] and can therefore be inhibited through a newly unearthed switch II pocket. The first approved therapy to ever directly target the KRAS oncoprotein in any KRAS mutant-mediated cancer was sotorasib, otherwise known as AMG510 [62].

Sotorasib is an orally active first-in-class irreversible covalent inhibitor designed to target the KRASG12C mutant in different cancer settings where these mutants are well-expressed. AMG510 assumes a stable bond conformation with the G12C mutated residue, therefore enforcing KRAS to remain in its inactive GDP-bound state and preventing its activation through the formation of its GTP form, which blocks the downstream signaling effects associated with cell growth, proliferation, and differentiation [62,63]. This covalent inhibitor gently switches the concentration of KRAS to its KRAS-GDP bound form with a half-life of 30 min, while that of the GTP-bound KRAS only stays for few seconds [64]. Other covalent inhibitors include MRTX849 (Mirati Therapeutics) and ARS-3248 (the new version of ARS-1620, Wellspring Biosciences and Jansen). While AMG510 covalently binds to the cysteine residue of the KRASG12C mutant, thereby locking KRAS in its inactive state [65], MRTX849 binds KRASG12C at the cysteine residues of position 12, thereby inhibiting the formation of the GTP-bound state of KRAS and blocking downstream KRAS-mediated signaling processes [66]. The other covalent inhibitor ARS-1620 can also inhibit KRAS at the G12C position. However, there have been challenges surrounding its suboptimal potency due to its low affinity for KRASG12C, and because a small portion of the pocket was occupied by ARS-1620 [67]. When researchers at Amgen collaborated with Carmot Therapeutics to screen for potent KRASG12C inhibitors, they found that several molecules bound within the pocket with diverse conformations. The crystallographic data of some of these compounds showed that a histidine residue could shift up to unravel a hidden groove, and the key breakthrough surrounding the emergence of AMG510 was that this groove created an alternative structural conformation of His95, which positioned it to occupy the aromatic rings of AMG510 and therefore enhanced KRASG12C protein interaction with the covalent inhibitor [68]. Further explorations into these mutant-binding compound mechanisms found that the best binders were able to flip off the histidine residue to struggle into the pocket of the mutant protein. The pharmacophoric relationship between AMG510 and ARS-1620 had been reported to have reduced due to the interaction of AMG510 with the His95 groove, which led to an approximately tenfold increment in AMG510 potency [69]. In a preclinical study presented by Amgen at the American Association for Cancer Research (AACR), administration of the KRASG12C inhibitor AMG510 in combination with the checkpoint inhibitor anti-PD-1 led to a complete remission of colon cancer in mice [32]. These data showed an initial upsurge in infiltrating T cells following AMG510 administration, further increased after anti-PD-1 administration. A pro-inflammatory microenvironment, characterized by upregulated interferon signaling, chemokine formation, antigen processing, and cytotoxic and natural killer cell activities, was observed [70]. The first human study (NCT 03600883) reported that 11 out of 23 NSCLC patients with locally advanced or metastatic KRASG12C mutant tumors had a partial response to AMG510 therapy. Likewise, in CLC patients and those with other tumors, 14 out of 19 achieved stable disease, even though no partial response was recorded. Adverse effects associated with AMG510 include gastrointestinal (GIT) side effects such as diarrhea and nausea [65].

Another orally available small molecule drug (identified through critical intensive structure-based drug design) with strong binding affinity for the KRASG12C mutant, MRTX849, inhibits the Ras/MAP kinase pathway by irreversibly binding to cysteine 12 in the inducible switch II pocket of KRASG12C, thereby blocking its conversion to the GTP-bound form and committing it to the GDP-bound state. MRTX849 possesses good linear pharmacokinetics (it is orally bioavailable) with extensive tissue distribution, which enhances smooth metabolism, absorption, and excretion with a half-life of 25 h after a single dose. Preclinical studies demonstrated its high KRAS-dependent signal transduction inhibitory potential and cancer cell viability, with EC50 of approximately 10nM. Besides this, it also expresses more than 1000-fold selective inhibition towards KRASG12C when compared with other cellular proteins. MRTX849 has displayed a wide spectrum of antitumor activity across several KRASG12C positive patients and cell-generated tumors, thereby promoting significant tumor reduction in most models and subsets of models. Pancreatic and lung cancer patient-derived models showed the most pronounced activity, while significant responses were also observed in KRAS mutant tumor models with co-mutations including STK11, KEAP1, and TP53 [67,71]. The pharmacokinetic attributes of MRTX849 predicted >30% human oral bioavailability with a half-life of approximately 20 h. The therapeutic index was estimated to be in folds of 10 during repeated administration trials to investigate the toxicity of this drug. Furthermore, its antitumor potential has been established for some other KRAS mutant-selective inhibitors, and it was the first molecule to hit IND-track development [67].

Other small molecule inhibitors with different mechanisms of action include luminespid (AUY922), tipifarnib (Zarnestra), AMG515 (Bimiralisib), KO-947, and LSZ102. AUY922 attacks heat shock protein-90 (HSP90), a chaperone protein, thereby inhibiting the stability and activation of KRAS, which might lead to its degradation and the blocking of its downstream signaling effects. Zarnestra, a farnesyltransferase inhibitor, inhibits the farnesylation (a process called prenylation, which involves the addition of lipid farnesyl group to proteins) of KRAS, thereby inhibiting the post-translational localization of KRAS to the cell membrane and preventing the initiation of downstream signaling pathways [72]. AUY922 binds to the ATP-binding site of the HSP90 chaperone responsible for the proper folding and stabilization of the KRAS oncogene, thereby disrupting its proper folding function, which leads to the destabilization and degradation of the KRAS mutant protein [73]. The inhibition of farnesyltransferase leads to the blockage of KRAS farnesylation and other proteins that rely on its modification for their activity. This resultantly prevents the proper localization and function of Ras in the cell membrane and ultimately inhibits the downstream signaling that promotes cell growth and survival. AMG510/AMG515, otherwise called bimiralisib, is a dual and simultaneous inhibitor of both KRAS and its downstream PI3K. It was developed to target the cross-talking between the KRAS and PI3K signaling pathways in cancer [74,75]. KO-947 targets the allosteric site of KRAS-GTP to disrupt the protein’s GTP-bound active state, thereby blocking its downstream signaling effects. Lastly, LSZ102 represents a selective inhibitor of wild-type and G12D mutant KRAS, but its precise mechanism of action is not fully understood [76].

Recently, the development of the KRASG12D inhibitory bicyclic peptide KS-58 was described, and proof of its anticancer potential against mouse patient derived xenografts (PDX) from a human pancreatic cancer cell line (PANC-1) harboring the KRASG12D mutation was presented. Furthermore, its anti-colorectal cancer potential was also shown in mouse tumors derived from a KRASG12D-mutant CLC CT26 cell line. The underlying mechanism includes the downstream inhibition of ERK phosphorylation, thereby inhibiting the cancer growth. It is imperative to note that KS-58 did not show synergistic anticancer potential with mouse anti-PD1, and morphological analysis with immunostaining results showed no differences in CD8^+^ T cell infiltration or PD-L1 expression levels in CT26-derived tumors when exposed to monotherapy or combination therapy. However, there was significant blood stability with an approximately 30 min half-life and no obvious systemic adverse effect [77]. Given the robust anticancer potential of KS-58 in CLC, repurposing of this drug for other cancers, like PDAC with KRASG12D mutation, may represent an efficacious alternative for their treatment.

Other drugs targeting the downstream proteins of KRAS mutation other than G12C have been challenging to develop due to the complexity of the RAS signaling pathway. However, some drugs have been designed and investigated to target downstream effectors, like RAF. This presents a viable approach as demonstrated by the success of selective BRAF-V600E inhibitors, including vemurafenib, encorafenib, and dabrafenib, which have all received FDA approval and shown significant clinical responses. However, these drugs are ineffective against RAS mutant tumors because of their limited inhibitory potential on dimerized RAF. Recently, IHMT-RAF-128, a highly potent pan-RAF inhibitor which has demonstrated inhibitory potential against both partners of RAF, was developed. This broad-spectrum inhibition is important as traditional selective inhibitors may not be effective against RAS mutant tumors due to their limited inhibitory potential on dimerized RAF. In addition, the pharmacokinetic assessment of this drug showed good bioavailability in mice and rats. Other studies showed that IHMT-RAF-128 demonstrated potent antitumor efficacy in xenograft mouse tumor models in a dose-dependent manner, without causing any apparent toxicities [78].

Further attempts to eradicate the difficulties encircling direct KRAS targeting have shown that single-agent inhibitory therapy, such as the use of MEK inhibitors, has not been effective in treating CLC. Therefore, a synergistic therapy that combines both trimetinib (MEK inhibitor) and vincristine to potentially treat mCRC (metastatic colorectal cancer) with KRAS mutations has been reported to be highly promising. This combinatory therapy has been found to be highly effective in cell growth inhibition, clonogenic survival reduction, and the promotion of apoptosis in multiple KRAS-mutant CLC cell lines. In addition, in KRAS-mutant PDX mouse models, this combination therapy has also demonstrated promising results, significantly inhibiting tumor growth, reducing cell proliferation, and increasing apoptosis in these models. The suggested mechanism underlying this synergistic effect was linked to the enhanced intracellular accumulation of vincristine, which is associated with MEK inhibition. This combination therapy was able to target many other downstream signaling pathways and protein components, including mTOR, which indicated the inhibition of both RAS-RAF-MEK and PI3K-Akt-mTOR signaling. The tolerance of the mice at doses of clinical significance suggests potential safety in humans [79]. This implies that combination therapy would definitely target more than a single signaling pathway, which presents a better therapeutic experience than monotherapy.

An attempt to develop a novel compound that could inhibit PDKs (pyruvate dehydrogenase kinases) as a potential therapeutic agent towards aggressive and resistant KRAS-mutant PDACs has been reported. PDKs are enzymes involved in cancer metabolism and have been implicated in cancer aggressiveness and chemoresistance mechanisms. Therefore, they represent molecular targets that should be investigated in further clinical trials for groundbreaking cancer drug discovery. Dicloroacetic acid (DCA) is the first PDK inhibitor to have entered phase II clinical trials. However, it has limitations related to its weak anticancer activity and the need for high doses, which results in several side effects. Following the development of a molecular hybridization approach devised to design and synthesize a small library of compounds called 3-amino-1,2,4-triazine derivatives, the PDK inhibitory potential of these compounds was reported. The capacity to induce cancer cell death at low micromolar doses, particularly against human pancreatic KRAS mutated cancer cells, was shown by these compounds. Further cellular studies to unravel the mechanism surrounding their anticancer potential revealed that these compounds disrupted the PDK/PDH axis, leading to metabolic and redox imbalance and ultimately triggering apoptotic cancer cell death. Furthermore, in the preliminary animal studies of a highly aggressive KRAS solid tumor model, it was demonstrated that the most representative compound effectively targeted the PDH/PDK axis in vivo with equal or even better efficacy, and it also had a better tolerability profile compared to the FDA-approved drugs cisplatin and gemcitabine. Therefore, it is strongly believed that the capacity of these compounds to target PDK, coupled with their non-toxic effect, qualify them as potential clinical candidates for combating highly aggressive KRAS-mutant PDACs. Further research and clinical trials will be necessary to validate these findings and potentially move these compounds towards clinical relevance [80].

## 4. Targeting Tumor Suppressor Mutations for PDAC Treatment

### 4.1. TP53

TP53 (*Trp53 in mice*) is a tumor suppressor gene that encodes p53, a protein that strongly prevents the initiation and progression of pancreatic cancer. It regulates physiological homeostasis via the induction of the expression of adjacent and distant genes that are critical to the normal growth of cells. However, p53 may repress the expression of other genes. The regulatory function of p53 varies under different conditions within the cell. Under unstressed conditions, cells maintain a low level of p53 through the E3 ligase mouse double minute 2 homolog (MDM2)-driven proteasome degradation of p53. MDM2 inhibits the activation of p53 and its regulatory function. The activation of p53 within a cell usually occurs in response to stress conditions. Endogenous stresses due to replication, oncogenic activation, hypoxia, and ROS, or exogenous stress due to nutrient deprivation, exposure to ionizing radiation, and cytotoxic agents culminate in p53 activation, induction of gene expression, apoptosis, autophagy, anti-vascularity, and maintenance of genetic stability [81,82]. An inactivating mutation in the TP53 gene can inactivate the tumor suppressive function of p53 protein. Mutation of the TP53 gene is recognized as one of the driver mutations for pancreatic cancer [81]. About 70% of PDAC patients harbor a missense mutation in the DNA-binding domain of p53 [83]. Mutant p53 (mut-p53) accumulates in PDAC patients, loses normal function, and becomes incapable of inducing the expression of the transcriptionally active p21, among other genes. This leads to an inability to arrest cell cycle progression. PDAC therefore continuously exploits the cell cycle without the p53-driven consequence of replication stress. When p53 loses normal function, it activates surrogate compensatory pathways that can promote tumor growth. Depending on cell types, mutation profiles, and epigenetics, the activities of certain molecules called synthetic partners are upregulated, and they sustain the incessant proliferation of PDAC in a phenomenon called synthetic lethality [83,84]. Mut-p53 proteins can also acquire a non-cell autonomous function that enables a favorable tumor microenvironment (TME) for PDAC to thrive. Cooks and colleagues have reported mut-p53-mediated reprogramming of tumor associated macrophages (TAMs) and enhanced TGF-β activities, leading to an anti-inflammatory and immunosuppressive TME [85]. In another study, mut-p53 induced the secretion of WNT ligands that stimulated the release of IL-1β by TAM and induced systemic neutrophilia and metastasis [86]. In cancer cells, mut-p53 contributes survival, proliferation, genome stability, metastatic potential, invasiveness, chemoresistance, metabolic rewiring, and the inhibition of autophagy and apoptosis [87,88,89,90,91,92].

#### 4.1.1. Targeting Negative Regulators of wt-p53

**Figure 2:**
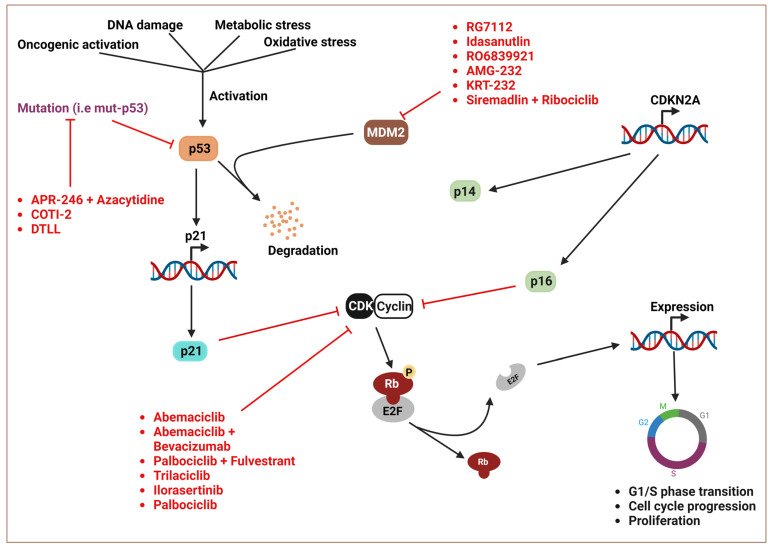
Shows p53, p16, and p14 tumor suppression pathways and targets of inhibitors.

Several approaches that can directly or indirectly target the negative regulators of p53 protein have been proposed (Figure 2; Table 2). In cancers with wild-type p53 (wt-p53), the expression of MDM2, a negative regulator of p53, is elevated; therefore, the inhibition of MDM2 has been an important therapeutic approach in preclinical and clinical studies. The cis-imidazoline derivatives, nutlins, were first identified in preclinical studies for their ability to disrupt the MDM2/wt-p53 interaction [93]. Following this, the efficacy of several nutlin derivatives and other MDM2 inhibitors has been investigated in clinical trials. However, most nutlin derivatives and other MDM2 inhibitors have been investigated only in phase 1 trials. RG7112 was the first selective MDM2 inhibitor that was investigated in clinical settings [94,95,96]. In a particular phase 1 trial, the administration of RG7112 to 20 patients with MDM-2-amplified, well-differentiated (WDLPS), or dedifferentiated (DDLPS) liposarcoma increased p53 concentration by a median of 4.86 times (IQR 4.86–7.97; *p* = 0.001), and MDM2 mRNA by a median of 3.03 times (1.23–4.93; *p* = 0.003) [97]. Mechanistically, RG7112 occupies the p53-binding pocket of MDM2, leading to p53 stabilization and activation [98].

Besides RG7112, several other nutlin-derived MDM2 inhibitors have been investigated. Idasanutlin was administered as a monotherapy to patients with polycythemia vera and essential thrombocythemia, and an overall response of 58% was recorded [99]. In a recent open label phase 1b two-dimensional dose-escalation study, the safety profile of idasanutlin was evaluated in combination with the BCL-2 inhibitor venetoclax in patients with r/r AML that were eligible for cytotoxic chemotherapy. Incidence rates for composite complete remission and morphologic leukemia-free state were 26% and 12%, respectively [100]. Currently, idasanutlin is being investigated in a phase 2 trial, and several phase 1 trials are now focusing on the pegylated prodrug of idasanutlin, RO6839921 [101]. Unlike idasanutlin, which has been primarily administered orally, RO6839921 was administered intravenously. This allowed plasma esterase to cleave the inactive prodrug to release the active idasanutlin, and the safety profile of RO6839921 was comparable with the oral idasanutlin [102].

In another phase 1b dose-escalation study, the MDM2 inhibitor siremadlin was administered in combination with the CDK4 inhibitor ribociclib to WDLPS or DDLPS patients. Three patients achieved partial response, while thirty-eight patients achieved stable disease. Although there were records of dose-limiting toxicities, such as hematological events, the combination therapy showed manageable toxicity and promising antitumor potential in patients with advanced WDLPS or DDLPS [103]. Most MDM2 inhibitors targeted towards advanced solid tumors have yielded at least a stable disease with mild treatment-related adverse events (TRAEs). When AMG-232 and KRT-232 were administered in separate phase 1 trials in patients with advanced solid tumors, no cardiac safety concern was noted in patients treated with KRT-232 [104], TRAEs were mild, and stable disease was achieved in AMG-232 treated patients [105]. In addition, the administration of AMG-232 and MDM-2 inhibitors like HDM201 leads to the elevation of p21, PUMA, BAX, MDM2, and the serum macrophage inhibitor cytokine-1 [106,107]. In a phase 1 trial, some MDM-2 inhibitors were shown to produce partial responses and moderate antitumor efficacy [108,109]. Taken together, the safety profile of MDM-2 inhibitors has been investigated in AML and advanced solid tumors. Based on the current evidence, MDM-2 inhibitors are promising targeted therapies for the treatment of PDAC.

#### 4.1.2. Targeting Mutant p53

Mut-p53 contains an unstable core that is highly vulnerable to loss of function mutations. While missense mutations represent the most prevalent mutation of this core, nonsense mutations of the TP53 gene are also known. In patients that harbor nonsense mutations, molecules that can promote p53 translation readthrough can be beneficial. These molecules can bypass the RNA stop codon to produce wt-p53 proteins. Aminoglycosides such as G418 and synthetic derivatives such as NB124 have been shown to rescue mut-p53 [110]. Although these types of molecules have not been evaluated in PDAC patients, phase III trial drugs like ataluren that increased translational readthrough in other diseases could be repurposed and evaluated for a similar role in PDAC patients.

In recent times, certain classes of small molecules that can bind and stabilize the mut-p53 core into a wild type-like conformation have been identified. These molecules can restore the tumor-suppressor function to mut-p53. Several mut-p53 targeting drugs have been developed. However, only two have been evaluated in clinical trials [82]. Strategies that have been used to target mut-p53 include stabilization of wt-p53, restoration of DNA-binding capacity to mut-p53, refolding, prevention of mut-p53 misfolding, and promoting the expression of full length wt-p53 from mRNAs with the nonsense mutation [82]. An example of an active clinical trial drug that can rescue mut-p53 and restore its tumor-suppressor function is the small molecule eprenetapopt (APR-246) [111]. The role of this drug was investigated in pancreatic cancer cell lines. It was found that APR-246 sensitized MIA-PACA-2 cells to berberine compounds [112]. Further, APR-246 has been investigated in several clinical trials. When APR-246 was administered in combination with azacytidine to mut-p53 patients with myelodysplastic syndrome, an overall response rate of 71% with 44% complete remission was achieved [113]. Mechanistically, APR-246 induces cancer cell apoptosis [114] and reprograms tumor-associated macrophages (TAMs) to enhance immune checkpoint inhibitors [115]. COTI-2, a thiosemicarbazone derivative, is another clinical trial drug that can target mut-p53. In several preclinical models, COTI-2 showed promising antitumor activities [116,117], and it is currently being evaluated in a clinical trial [118].

### 4.2. SMAD4

Smad4, also called ‘deleted in pancreatic cancer 4 (DPC4)’, was the first discovered member of the Smads family. Other members of the Smad family were isolated in mid-1990 from *Drosophila melanogaster.* The Smad4 gene occupies the chromosome locus 18q21.1 [119]. In pancreatic patients, there is a homozygous deletion of the Smad4 gene in about 30% of the patients, and an allelic loss in another 20% [120]. Overall, approximately 55% of PDAC patients harbor inactivation or loss of the Smad4 gene [121]. Smad4 is a tumor suppressor protein that plays a role in the TGFβ signaling pathway [122]. The role of the TGFβ signaling pathway in cancer development is paradoxical. One axis that depends on Smad4, called the Smad4-dependent axis of the TGFβ pathway, has been shown to autonomously mediate tumor suppression via the induction of Smad4-controlled genes, cell cycle arrest, and apoptosis [122,123]. However, when SMAD4 loses normal function due to missense, nonsense, or frameshift mutations, the canonical TGFβ/Smad4 signaling axis becomes abrogated and the tumor suppression functions are counteracted. This loss of function is compensated by Smad4-independent TGFβ pathways, including the PI3/Akt, Ras/Erk, MSP/RON, STAT3, and Nf-кβ/PTEN pathways, leading to invasiveness and aggressive tumor progression [122,124]. 

The status of Smad4 in cells has prognostic significance [125]. A plethora of studies have investigated the prognostic value of Smad4 in various diseases. Loss of Smad4 is associated with worse overall survival and disease-free survival [126]. Epithelial to mesenchymal transition (EMT) promotes pancreatic cancer progression, and the loss of Smad4 promotes EMT and increases resistance to therapies [127]. In a randomized multicentered phase III CONKO-005 trial, targeted sequencing and expression analysis revealed five patient clusters. In one, multivariable Cox regression analysis showed that Smad4 aberration was negatively associated with gemcitabine response. However, erlotinib was shown to counteract the effect of mutant Smad4, and thus promote the response to gemcitabine treatment [128]. This suggests that erlotinib may target aberrant Smad4 to promote a therapeutic response. Given the significance and frequency of aberrant Smad4 mutations in PDAC, drugs that can restore mutant Smad4 function are inevitable. Unfortunately, aberrant Smad4 is one of the undruggable targets in PDAC patients. To date, there is no standard-of-use drug or new investigational drug that can target mutant Smad4. However, several studies in clinical trials have shown that some chemotherapy and immunotherapy may be beneficial to PDAC patients with Smad4 mutations, but it is unknown whether such therapies specifically target Smad4. Further, several preclinical studies have identified promising agents that could target mutant Smad4 and restore its function. The use of dual-targeting ligand-based lidamycin (DTLL) not only inhibited tumor progression by blocking the ATR/mTOR pathway, but also restored Smad4-mediated activation of Nf-кβ shunt, leading to significant remission in chemoresistant PDAC [129]. The practice of folk medicine is not dying out in the modern world [130]. When patients with benign prostatic hyperplasia were treated with the Chinese medicinal compound qianlongtong (qlt), plasma containing qlt was collected and supplemented to prostate stromal cells for 24 h. PCR and Wb-based analysis of Smad4 expression revealed that qlt-containing plasma inhibited proliferation and enhanced apoptosis in the prostate stromal cells by increasing the expression of Smad4 [131]. The study suggests that qlt may be an activator of Smad4 in prostate stromal cells. Thus, the evaluation of qlt or its derivatives in a PDAC preclinical model may yield promising results. Myhre syndrome (MS) is driven by pathogenic variants of Smad4 gene. Losartan improves skin thickness and joint range motion in MS [132]. The beneficial role of losartan in Smad4-deficient diseases like MS suggests that losartan may indirectly remediate aberrant Smad4. 

Due to limited direct Smad4 inhibitors, the selective inhibition of the surrogate compensatory TGFβ-pathways may be favorable for PDAC patients (Table 2). The TGFβ-PI3/AKT axis is activated in many chemoresistant tumors. Matched therapy that targets the PI3/AKT/mTOR axis provides significant clinical benefits [133]. Many antagonists of this pathway have been evaluated in clinical trials. The safety profile of duvelisib was evaluated in a phase I trial involving T-cell lymphoma patients [134]. Several antagonists of the PI3/AKT axis passed phase I and are being investigated in subsequent phases. The efficacy of perifosine and MK-2206 has been investigated in phase II trials for recurrent glioblastoma and advanced breast cancer, respectively [135,136]. Further, targeting other TGFβ-activated pathways like the MSP/RON pathway may be promising [137]. Taken together, PDAC patients may benefit from targeting other downstream pathways of TGFβ.

### 4.3. CDKN2A

The CDKN2A gene encodes the p16 (p16^INK4A^) and p14 (p14^ARF^) tumor suppressor proteins. It is adjacent to the CDKN2B gene on locus 9p21. The p16 and p14 tumor suppressor proteins are transcribed from different first exons—exon 1α, p16; exon 1β, p14—but share exons 2 and 3 [138]. After the first discovery of cyclin-dependent kinase (CDK) inhibitors in 1993 [139], the role of p16, a CDK inhibitor in cell cycle regulation, has been established. The p16 protein inhibits CDK4 and CDK6 activities, thus preventing the inactivating phosphorylation of the RB protein, culminating in senescence and cell cycle arrest [140]. Further, the second tumor suppressor protein p14, encoded by the CDKN2A gene, promotes the activities of p53 protein by inhibiting the MDM2-mediated proteasome degradation of p53 [141]. However, somatic mutations, deletions, and promoter hypermethylation culminate in loss of CDKN2A function. The inactivation of CDKN2A is associated with the development of colorectal cancer [142], melanoma [143], brain tumors [144], mesotheliomas [145], meningiomas [146], and so on. In pancreatic cancer, somatic loss of the CDKN2A gene drives pancreatic tumorigenesis. Besides melanoma, the CDKN2A pathogenic germline variant also promotes predisposition to pancreatic cancer. This pathogenic variant has been identified in more than 3% of PDAC patients, which contributes a 12.3-fold increased risk of developing pancreatic cancer [147].

Drugs that can directly restore the tumor suppressor function to defunct/mutated CDKN2A are unavailable. Therapeutic strategies have focused on developing agents that can mimic the CDK4/6 inhibition activities of p16 protein (Table 2; Figure 2). To this end, most CDK4/6 inhibitors developed have been used in combination therapy to treat advanced breast cancer (ABC). Abemaciclib is an oral, continuously dosed CDK4/6 inhibitor approved for the treatment of ABC. The efficacy of abemaciclib was evaluated in an adjuvant setting, specifically in an open-label phase III trial in patients with HR+, HER2-, node-positive early breast cancer. Among 5637 randomly assigned patients, the combination of abemaciclib and endocrine therapy offered a superior IDFS compared to endocrine therapy alone (*p* = 0.01, hazard ratio, 0.75; 95% CI, 0.60 to 0.93) [148]. In a placebo-controlled randomized trial (NCT01942135), the CDK4/6 inhibitor Palbociclib plus fulvestrant or placebo-fulvestrant were randomly administered to 512 patients with HR+, HER2− ABC who had sensitivity to previous endocrine therapy. Patients that received combination therapy had improved median overall survival of 34.9 months (95% CI, 28.8 to 40.0) compared to 28 months (95%, 23.6 to 34.6) in patients that received placebo-fulvestrant [149]. As these findings have shown that CDK4/6 inhibitors are promising therapies for the treatment of ABC, we advocate that CDK4/6 inhibitors be repurposed for pancreatic cancer.

The safety and efficacy of CDK4/6 inhibitors have also been evaluated in other diseases. Abemaciclib combination therapy yielded promising results in ABC, and the combination of abemaciclib and bevacizumab is currently being evaluated in recurrent GBM patients with heterozygous or homozygous loss of CDKN2A (NCT04074785). Phase Ib open label and phase II, randomized, double-blind placebo-controlled trials evaluated the safety and efficacy of trilaciclib—an intravenous CDK4/6 inhibitor—in treatment-naïve extensive-stage small-cell lung cancer. The combination of trilaciclib with etoposide/carboplatin were well tolerated. There was evidence of myelopreservation and improved antitumor efficacy. In preclinical models, trilaciclib preserved hematopoietic stem and progenitor cells (HSPC) by maintaining HSPC in G1 arrest, enhancing their quick recovery, and boosting antitumor immunity [150]. In many CDNK2A deficient hematological cancers and solid tumors, the efficacy and pharmacokinetics of the multikinase inhibitor ilorasertinib were evaluated [151,152]. Many studies involving ilorasertinib have been phase I pilot studies. However, a phase II study that involved 12 patients with advanced solid tumors was terminated due to five adverse events including vomiting, uncontrolled pain, sepsis, cervical stenosis, and hypertension (NCT02478320). The role of CDK4/6 inhibitors in therapeutic approaches suggests that combination therapy that includes CDK4/6 inhibitors could offer superior clinical benefits in patients with solid tumors. However, only a few studies have evaluated the therapeutic efficacy of CDK4/6 inhibitors in pancreatic cancer. In a non-randomized, open label, phase II study involving 19 patients with metastatic Grade 1 and 2 pancreatic neuroendocrine tumors, the administration of palbociclib did not yield any objective response. Eleven patients (57.9%) had stable disease. The median PFS was 2.6 months (95%, 0 to 14.4), and the median OS was 18.7 months (95%, 7.4 to 29.9). However, treatment related toxicities, including asthenia (76.2%), neutropenia (42.9%), diarrhea (33.3%), and nausea (33.3%) were observed [153].

**Table 2:** Inhibitors and their targets in various aberrant tumor suppressor pathways.

Target	Agent(s)	Phase	Disease(s)	Mechanism	Reference(s)
MDM2	RG7112	I	WDLPS/DDLPS	Occupy p53 binding pocket of MDM2 for p53 stabilization/activation	[94,95,96,97,98]
MDM2	Idasanutlin	I	Polycythemia Vera and Essential Thrombocythemia	Promote TP53 expression and prevent p53 degradation	[99]
MDM2	Venetoclax-Idasanutlin	Ib	Relapsed/Refractory Acute Myeloid Leukemia	p53 activation to inactivate MCL-1 and BCL-xL	[100]
MDM2	RO6839921	I	Acute Myeloid Leukemia	Plasma esterase cleaves inactive prodrug to release active Idasanutlin to restore p53 activity	[101,102]
MDM2 and CDK4/6	Siremadlin + Ribociclib	Ib	WDLPS/DDLPS	p53 pathway activation and inhibition of CDK enzymes	[103]
MDM2	AMG-232	I	P53WT Solid Tumors or Multiple Myeloma	Strong binding blocking MDM2-p53 interaction	[105,106,107]
MDM2	KRT-232	I	Solid Tumors or Multiple Myeloma and Acute Myeloid Leukemia	Binds to MDM2 and inhibits interaction with p53 for p53 activation	[104]
Mutant p53	APR-246 + azacytidine	Ib/II	Myelodysplastic Syndrome	Induce apoptosis and reprogram TAMS to enhance immune checkpoint inhibitors	[113]
Mutant p53	COTI-2	I	Head and Neck Squamous Cell Carcinoma and Gynecologic Malignancies	Restore functionality to mutated p53	[118]
SMAD4	DTLL	Pre-Clinical	PDAC	Block ATR/mTOR pathway and restore SMAD4 mediated activation of Nf-кβ shunt	[129]
SMAD4	Qianlongtong	Randomized Control	Benign Prostate Hyperplasia	Increase expression of SMAD4	[131]
SMAD4	Duvelisib	I	T-cell Lymphoma	PI3K-δ/γ inhibition in TGFβ-PI3/AKT Axis	[134]
SMAD4	Perifosine and MK-2206	II	Recurrent Glioblastoma and Breast Cancer	MSP/RON pathway in TGFβ-PI3/AKT Axis	[135,136]
CDK4/6	Abemaciclib	Open label III	HR+, HER2-, node-positive, Breast Cancer	Inhibition of cell cycle progression through CDK4/6 inhibitor	[148]
CDK4/6	Abemaciclib + Bevacizumab	I	Recurrent GBM with loss of CDKN2A	Inhibition of cell cycle progression through CDK4/6 inhibitor and anti-angiogenic therapy	NCT04074785
CDK4/6	Palbociclib + Fulvestrant	Placebo Controlled Randomized Trial	HR+, HER2-, Breast Cancer	Inhibition cell cycle progression through CDK4/6 inhibitor	NCT01942135
CDK4/6	Trilaciclib	Ib/II	Naïve Extensive Stage Small Cell Lung Cancer	Chemotherapy damage prevention by HSPC remaining in G1 arrest	[150]
CDNK2A	Ilorasertinib	II	Advanced Solid Tumors	Multikinase Inhibition to induce cell cycle arrest	NCT02478320
CDK4/6	Palbociclib	Non-randomized Open Label II	Metastatic Grade 1 and 2 Pancreatic Neuroendocrine Tumors	Inhibition of cell cycle progression in RB+ cells through CDK4/6 inhibitor	[153]

## 5. Conclusions

Pancreatic cancer is one of the leading causes of cancer-related death. Several therapeutic approaches have offered minimal clinical benefits. In the last 5 decades, the 5-year overall survival rate of pancreatic cancer patients has only increased by 10%. While this represents significant progress in the field, the estimated mortality of pancreatic cancer patients has continued to increase. Surgery and chemotherapy are standard-of-use approaches for the treatment of PDAC. Up to 20% of PDAC patients have resectable tumors and could therefore benefit from surgery [154]. Some of this patient population usually have low-stage tumors, and therefore survive longer than 5 years compared to those with an advanced stage of the disease. Sadly, low-tumor stage diagnosis is incidental and comprises only up to 5% of the cases [155]. Combination therapies like FOLFIRINOX, or combinations with radiotherapy, have been used as neoadjuvants to shrink tumors prior to surgery in borderline resectable tumors. They have also been used as adjuvant therapies to prevent postoperative relapse. In other populations, surgery is not beneficial due to metastasis and the general presence of high-stage tumors. In non-resectable or metastatic advanced PDAC, a combination of chemotherapy and radiotherapy is the available treatment option. The success of chemotherapy has been challenged given the absence of actionable targets, as the biological nature of PDAC makes it impenetrable for most chemotherapy agents. In addition, many agents are highly expensive and do not provide durable clinical benefits to PDAC patients. 

In the context of genomic drivers of PDAC, the attention of the field has been drawn to the identification of actionable targets and the development of robust targeted therapies. Driver mutations in oncogenic KRAS and the tumor suppressors TP53, CDKN2A, and SMAD4 genes represent actionable drug targets. The development of therapies against these targets has been challenging. For instance, the FDA approval of sotorasib for targeting the KRASG12C mutation in PDAC patients has been beneficial, but only for a limited patient population, because the KRASG12C mutation is only found in less than 2% of PDAC cases. Despite efforts to identify several molecules that can target these drivers, many of these drugs have not been approved. Several molecules that showed promising preclinical efficacy were not successful in clinical trials due to intolerability at low doses, treatment-related adverseness, failed objective response, and an inability to compete with the currently approved anticancer drugs. Although there are records of failed disease remission in clinical trials, it is important to stress the promising efficacy of other targeted therapies both in clinical trials and upon approval. Such impressive outcomes necessitate more research in the area of druggable target identification and the development of target-specific therapies.

## Figures and Tables

**Table 1:** Some of the most frequently mutated genes.

S/No	Gene	Frequency of Mutation in PDACs	Types of Mutation	Amino Acid Residues	References
1	KRAS	90%	Missense point	G12C, G12D, and G12V	[28,29,30,31]
2	CDNK2A	30–40%	Point mutation, deletion and loss of heterozygousity, insertion and frame shift mutation, promoter methylation, splice site mutation	P16INK4A (p16-Leu148) and P14ARF mutations	[32,33]
3	DPC4/SMAD4	50%	Nonsense mutation, missense mutation, frameshift mutation, splice site mutation, deletion and insertion, point mutation, promoter methylation, large rearrangement, silent mutation	MH1 domain: R361C, R361H, R361S, and R361G MH2 domain: R100C, D351N, L384P, P529L. Linker region: E249K, G253V C-terminal region: Q408P and G437E	[19,32]
4	TP53	≥50%	Missense, nonsense, frameshift, splice site, deletion and insertion, promoter methylation, hotspot, wild-type p53.	R17H, R28Q, R273H, R282W, and Y220C	[19,34]
